# An accessory enzymatic system of cellulase for simultaneous saccharification and co-fermentation

**DOI:** 10.1186/s40643-022-00585-5

**Published:** 2022-09-19

**Authors:** Han Liu, Xuxin Wang, Yanping Liu, Zhuoran Kang, Jiaqi Lu, Yutong Ye, Zhipeng Wang, Xinshu Zhuang, Shen Tian

**Affiliations:** 1 College of Life Science, Capital Normal University, Beijing, 100048 China; 2 Department of Environmental Science and Engineering, Beijing University of Chemical Technology, Beijing, 100029 China; 3 Guangzhou Institute of Energy Resources, Chinese Academy of Sciences, Guangzhou, 510640 China

**Keywords:** Hemicellulosome, Simultaneous saccharification and co-fermentation, Cellulosic ethanol, Consolidated bioprocessing, *Saccharomyces cerevisiae*

## Abstract

The enhanced hydrolysis of xylan-type hemicellulose is important to maximize ethanol production yield and substrate utilization rate in lignocellulose-based simultaneous saccharification and co-fermentation system. In this study, we conduct δ-integration CRISPR Cas9 to achieve multicopy chromosomal integration with high efficiency of reductase–xylitol dehydrogenase pathway in *Saccharomyces cerevisiae*. Subsequently, we devise a consolidated bioprocessing-enabling *S. cerevisiae* consortium, in which every engineered yeast strain could secrete or display different assembly components to be adaptively assembled on the surface of scaffoldin-displaying yeast cell for synergistic catalysis and co-fermentation from steam-exploded *Pennisetum purpureum*. Despite the accumulation of xylitol, the maximum ethanol titer of the genetically engineered yeast strain reached 12.88 g/l with the cellulose conversion of 91.21% and hemicellulose conversion of 55.25% under 30 ºC after 96 h with the addition of commercial cellulase. The elaborated cellulosomal organization toward genetic engineering of an industrially important microorganism presents a designed approach for advanced lignocellulolytic potential and improved capability of biofuel processing.

**Graphical Abstract:**

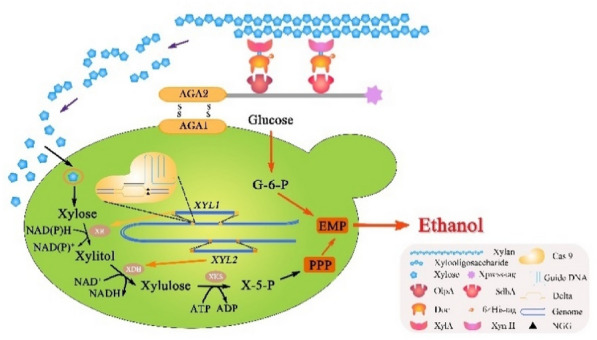

**Supplementary Information:**

The online version contains supplementary material available at 10.1186/s40643-022-00585-5.

## Keypoints

1. A replicative and integrative Di-CRISPR platform was promising in generating an efficient xylose-utilizing *Saccharomyces cerevisiae* strain.
2. The functional activity of chimeric xylanases was demonstrated in direct hemicellulose-to-ethanol conversion.
3. The feasibility of an accessory enzymatic system of cellulase for simultaneous saccharification and co-fermentation from pretreated C_4_ grass was evaluated.

**Supplementary Information:**

The online version contains supplementary material available at 10.1186/s40643-022-00585-5.

## Introduction

Second-generation bioethanol, produced by available sugars in the renewable lignocellulosic feedstock, is a promising alternative biofuel due to the diversity and abundance of available resources. It is well-known that the perennial C4 grass is a sustainable feedstock for biofuel production, since it can avoid competition with food crops because of its high photosynthesis ability and extensive environmental adaptability (Tubeileh et al. 2016). However, one of the main bottlenecks in achieving a competitive and economically feasible lignocellulose-based bio-refinery with broadened productive character is the current technological impediments to improving production yield and bioconversion efficiency.

Co-fermentation of pentose and hexose through the simultaneous saccharification and microbial metabolism process of biomass is realized to be a significantly important strategy for a competitive lignocellulosic biorefinery and is economically feasible. *Saccharomyces cerevisiae* is the classic industrial microorganism with a native capacity to produce ethanol. Thus, it is generally utilized as the ideal host cell for engineering genetic strain to perform the simultaneous assimilation of glucose and xylose (Wang et al. 2018). So far, efficient biofuel conversion for meeting the needs of industrial application from lignocellulosic materials remains a challenge despite many efforts that have been made for process optimization and integration in biomass-refinery. Few reports have demonstrated an ideal bioconversion rate based on holocellulose (cellulose + hemicellulose).

Consequently, the efficient hydrolysis of xylan-type hemicellulose has been a significant focus in S. cerevisiae strain-engineering of simultaneous saccharification and co-fermentation (SSCF) (Hoang et al. 2020) for maximizing both utilization rate and production yield. Xylan, the main hemicellulose component of biomass, is a polysaccharide formed by units of xylose as the backbone with β-1,4 linkages, partially substituted with arabinose, uronic acids, and acetyl groups, which can be hydrolysed into xylooligosaccharides and xylose by xylanases and xylosidases (Bhattacharya et al. 2015; Polizeli et al. 2005). These hydrolytic products, however, cannot be metabolized by the wildtype *S. cerevisiae*. Therefore, a promising recognition of consolidated bioprocessing (CBP) for whole-cell catalysis, which combines different functional enzymes of xylan-degrading and xylose-assimilating in one yeast strain, emerges as a novel configuration for the SSCF process for direct cellulosic ethanol bioconversion (Park et al. 2020; Liu et al. 2019). CBP-microorganism could be described as a sustainable biocatalyst that presents higher catalytic efficiency and better substrate selectivity due to its utility in cofactor regeneration, and multistep reactions can be carried out in a single strain under milder operational conditions. As one of the most robust and intricate molecular nanomachines, cellulosomes facilitate synergistic activity and enhance close proximity among different enzymatic systems, which enhances the hydrolysis of lignocellulose (Smith et al. 2013). Hence, cellulosome is required as an available resource for constructing CBP-enabling microbial strain. Several developments of engineered cellulolytic complex attaching with cellulosomal enzymes have been reported (Goyal et al. 2011; Sun et al. 2012; Tsai et al. 2013). However, “chimeric hemicellulases and mini-hemicellulosome” have few been employed in the “arming yeast” strategy for simultaneous hydrolysis and co-fermentation of pentose and hexose.

In this work, to construct the engineered *S. cerevisiae* with enhanced xylose catabolism by introducing the xylose reductase (XR)–xylitol dehydrogenase (XDH) pathway, we harnessed Di-CRISPR (Delta-integration CRISPR–Cas9) to specifically generate double-strand breaks at the δ-sites in yeast genome to increase the recombination efficiency of *XYL1* and *XYL2,* encoding for xylose reductase and xylitol dehydrogenase, respectively. Based on the construction of xylose-utilizing *S. cerevisiae* strain, the aim of the present study was: (1) demonstrate the potential of xylose-fermenting *S. cerevisiae* as the host for the engineering of synthetic minicellulosome attaching with xylan-hydrolysing enzymes together with the heterologous expression of XR and XDH; (2) evaluate the efficiency of this lignocellulose-based CBP system of simultaneous saccharification and co-fermentation in terms of holocellulose bioconversion from pretreated *Pennisetum purpureum*, a perennial C4 grass.

## Materials and methods

### Raw materials

The *Pennisetum purpureum* was obtained from the Guangzhou Institute of Energy Conversion, CAS. The materials were pretreated by steam explosion at 1.6Mpa and 195 °C for 5 min (Institute of Process Engineering, CAS). The pretreatment material was used as SSCF substrate without detoxification. The major chemical composition including lignin and structural carbohydrates of treated and untreated grass was summarized (Table 1) and analyzed following NREL standard analytical procedure (TP-510-42,618).

**Table 1:** Main chemical compositions of untreated and treated *Pennisetum purpureum* (% of DM)

Materials	Cellulose (glucan)	Hemicellulose (xylan and arabinan)	Lignin^b^	Ash	Extractives + Others^c^
Unpretreated^a^	35.2 ± 0.38	25.0 ± 3.74	20.1 ± 1.33	7.5 ± 0.20	12.2 ± 0.21
Pretreated^a^	48.7 ± 0.41	8.3 ± 0.19	29.4 ± 0.38	8.2 ± 0.01	5.4 ± 0.30

All values are averages ± standard deviation of three independent experiments

^a^Data shown as percentage of dry Matter (DM)

^b^Acid-soluble lignin included

^c^Non-structural material and other compounds from biomass

### Strains and media

*S. cerevisiae* strain WA1 was used as the host for xylose metabolic pathway engineering. The xylose-utilizing strain was then used to display adaptor scaffoldin and assemble minicellulosome architecture. The strains and plasmids used in this study are listed in Table 2. Yeasts were routinely maintained in yeast extract peptone dextrose (YPD) medium containing 2% glucose. All yeast transformants were selected and maintained on a synthetic dropout nutrient medium (SD-Ura/-Trp). *Aspergillus oryzae* 2120 and *Trichoderma reesei* 40,358 were purchased from the China Center of Industrial Culture Collection. *Clostridium thermocellum* ATCC 27,405 was purchased from DSMZ (Germany). All DNA manipulations were performed in *Escherichia coli* DH5α, which was grown in Luria Bertani (LB) broth with 10 mg/L ampicillin.

**Table 2:** Summary of the recombinant *S. cerevisiae* strains used in this study

Plasmids or Strain	Genotype/property	Resource
S. cerevisiae		
W303-1A	*MAT*a *ade2 trp1 his3 can1 ura3 leu2*	
WA1	W303-1A derivative, {PGK1p-Aga1-Leu2-Matt}, integrated into the chromosome expressing Aga1 in cell surface	Tian, 2019
WA1^δ^/XR-XDH	WA1 derivative, {PGK1p-*XYL1*-Matt; PGK1p-*XYL2*-Matt}, based on traditional delta integration	This work
WA1^g^/XR-XDH	WA1 derivative, {PGK1p-*XYL1*-Matt; PGK1p-*XYL2*-Matt}, based on delta integration enhanced by CRISPR/Cas9	This work
WA1^g^/ScafI	WA1^g^/XR-XDH derivative, surface displaying the achoring scaffoldin ScafI	This work
WA1^g^/XA	WA1^g^/XR-XDH derivative, expression of catalytical module XylA containg dockerin OlpA	This work
WA1^g^/Xn	WA1^g^/XR-XDH derivative, expression of catalytical module XynII containg dockerin SdbA	This work
WA1^g^/NC	WA1^g^/XR-XDH derivative, was transferred plasmid PYD1 as control	This work
Plasmids		
YD1	Ori, GAL1p, Aga2, Matt, Amp, Trp3	Commercial
pYD1–PGK–aga2	pYD1, PGK1p, Aga2, Matt, Amp, Trp3	Tian, 2019
pYD1–PGK–αMF	pYD1, PGK1p, αMF, Matt, Amp, Trp3	Tian, 2019
pCRCT	Ura3, encoding iCas9, tracrRNA and crRNAs	Commercial
pDi-g1	pCRCT, gRNA targeting δ sequence	This work
pYD1–ScafI	pYD1, PGK1p-Aga2-*ScafI*-Matt, Amp, Trp3	This work
pYD1–XylA	pYD1, PGK1p-αMF-*XylA*-*doc*-*OlpA*-Matt, Amp, Trp3	This work
pYD1–XynII	pYD1, PGK1p-αMF-*XynII*-*doc*-*SdbA*-Matt, Amp, Trp3	This work

#### DNA manipulation and plasmid construction

The recombination plasmid pDi-g1 for δ-integration CRISPR–Cas9 was designed and modified from the pCRCT plasmid (Beijing Zoman Biotechnology Co., Ltd) with specific gRNA, which mediates iCas9 protein targeting the delta sequence to generate specific double stranded breaks (DSBs). The gRNA1-F/gRNA1-R was incubated to form duplex DNA encoding the specific gRNA targeted delta sequence. The gRNA was inserted into the *Bsa*I site of pCRCT to generate pDi-g1.

The *XYL1* (NCBI: X59465) and XYL2 (NCBI: XM_001386945) were obtained from the genomic DNA of *P. stipitis* CBS 6054. 5’δ and 3’δ are fragments of the delta sequence, both of them and PGK1p were obtained from genomic DNA of *S. cerevisiae* W303-1A. Matt was amplified from the plasmid pYD1. *XYL1* and *XYL2* expression cassettes were individually over-lap PCR amplified with homologous arms and purified (Universal DNA pure Kit, BGI, China).

For δ-integration CRISPR–Cas9, 300 ng each of donor DNA was mixed with 1 μg pDi-g1 and transformed into WA1 to generate WA1^g^/XR-XDH strain. The same operation was carried out in the control group for conventional δ-integrated assembly via donor DNA fragments without pDi-g1 to generate a control strain WA1^δ^/XR-XDH. More details about plasmid construction were summarized in the supplementary information.

The DNA encoding two specific cohesin domains of SdbA (NCBI: AAB07763.1, residues 29-238) and OlpA (NCBI: Q06848.1, residues 30-180) were amplified by PCR from *Clostridium thermocellum* ATCC 27,405. The two DNA sequences were spliced into a fusion gene fragment *ScafI* by over-lap PCR. *ScafI* was digested with *BamH*I and *Not*I and cloned into the 3’-terminal of *Aga2* gene in plasmid pYD1–PGK–aga2 to generate the cell-surface displaying plasmid of anchoring scaffoldin. The anchoring scaffoldin ScafI was labeled with a C-terminal Xpress tag. The genes of the xylosidase (*XylA*, NCBI: AB013851.1) of *A. oryzae* and the endoxylanase (*XynII*, NCBI: U24191.1) of *Trichoderma reesei* were 3ʹ-terminal fused with the *doc*-*OlpA* and *doc*-*SdbA*, respectively. Then they were digested and cloned into pYD1–PGK–αMF for constructing xylanases secretion-expressed plasmids pYD1–XylA and pYD1–XynII. Both DNA fragments of *XynII*-*doc*-*SdbA* and *XylA*-*doc*-*OlpA* were designed to contain a C-terminal 6 × His tag for protein purification.

The plasmids of pYD1–ScafI, pYD1–XylA, and pYD1–XynII were, respectively, transformed into WA1^g^/XR-XDH to obtain recombination strain WA1^g^/ScafI, WA1^g^/XA, and WA1^g^/Xn. The yeast transformations were performed using the standard lithium acetate procedure. All recombinant yeast strains used in this study are listed in Table 2.

### Copy number determination of XR-XDH pathway

The integrated gene copy numbers of recombinant strains were determined by real-time quantitative PCR (RT-qPCR) using the yeast genomic DNA. Yeast cells were collected from an overnight culture and genomes were extracted from 2 × 10^7^ cells. The *XYL1*, *XYL2* of site-specific integration and *S. cerevisiae ALG9* gene were chosen as the target gene and reference gene, respectively. The plasmid containing the reference gene or target gene was used to generate the standard curves. The relative copy numbers of the target genes were determined by comparing them with the reference gene (Shi et al. 2016). Real-time qPCR reactions were performed using *PerfectStart*^®^ Green qPCR SuperMix (TransGen Biotech, China) on a BioRad CFX96 Touch real time PCR system.

### Enzyme assay

The WA1^g^/XA and WA1^g^/Xn were severally grown in YPD medium at 30 °C for 48 h before centrifuging the cultures at 4000 rpm for 5 min at 4 °C. The supernatant was used to determine the activity of the two enzymes. Endoxylanase activity was determined by dinitrosalicylic acid (DNS) method (Wood et al. 1988). Add the supernatant into the reaction mixture containing 50 mM Tris–HCl buffer (pH 5.5) and 0.5% specified substrate at 45 °C for 20 min, then add DNS and boil for 10 min. The concentration of xylose was measured by detecting the absorbance value of enzyme reaction mixture at 540 nm. The amount of enzyme required to produce 1 mg xylose per hour under the above conditions is defined as an enzyme-activity unit. Xylosidase activity was measured in 50 mM sodium citrate buffer (pH 5.0) with 5 mM 4-nitrophenyl β-D-xylopyranoside (*p*NPX) as substrate for 10 min at 30 °C. One unit of enzyme activity is defined as 1 μM liberated sugar from substrate per minute by measuring at OD_405 nm_ by UV–Vis spectrophotometer (Nanmori et al. 1990).

Cell-free extracts for xylose metabolic enzyme determination were prepared as follows. The recombinant xylose-utilizing yeasts WA1^δ^/XR-XDH and WA1^g^/XR-XDH were cultured in YPD medium containing 2% glucose for 48 h at 30 °C. Cells were collected by centrifuging at 4000 rpm for 5 min and washed twice with PBS buffer. They were then resuspended in 4 ml cold PBS and disrupted by ultrasonic disruption for 15 min. Centrifuging the cell lysates at 4000 rpm for 25 min and supernatants were collected for activities determination of XR and XDH. Protein concentrations of cell-free extracts were detected using the BCA Protein Assay Kit (Beyotime, Shanghai, China). XR activity was measured by monitoring the change of NADPH at 340 nm in 100 mM sodium phosphate buffer (pH 7.0) containing 0.15 mM NADPH and 200 mM D-xylose. XDH activity was determined by monitoring the production of NADH at 340 nm in 100 mM Tris–HCl buffer (pH 7.0) with 1 mM MgCl_2_, 50 mM xylitol, and 5 mM NAD^+^. The specific activities of the enzymes were expressed as micromoles of converted NADPH/NAD^+^ per minute per milligram of protein (U/mg).

### Protein purification and SDS–PAGE analysis

The recombinant yeast WA1^g^/Xn and WA1^g^/XA were incubated at 30 °C for 48 h. Cell culture supernatants were collected and centrifuged at 4000 rpm for 15 min. The supernatants after centrifugation and binding buffer (20 mM imidazole, 20 mM phosphate buffer, 200 mM NaCl) were loaded into the Ni–NTA column (Nanomicro Technology, China) in a ratio of 1:2 (v/v). Then, proteins were washed by gravity flow with 150 ml wash buffer (30 mM imidazole, 20 mM phosphate buffer, 200 mM NaCl) before being eluted with 30 ml of elution buffer (200 mM imidazole, 20 mM phosphate buffer, 200 mM NaCl). Each 20 µl of purified supernatant was mixed with 5 × Loading buffer and analyzed by SDS–PAGE.

### Immunofluorescence microscopy and flow cytometry

The culture of yeast expressing ScafI and enzyme at 24 h was centrifuged for 10 min at 3000 r/min at 4 °C. After centrifugation, WA1^g^/ScafI cells were retained and re-suspended with buffer (50 mM Tris–HCl, 100 mM NaCl, 10 mM CaCl_2_), while the supernatant of WA1^g^/XA and WA1^g^/Xn were retained. The two hemicellulases were, respectively, incubated with WA1^g^/ScafI cell suspensions or co-incubated with WA1^g^/ScafI in equal proportion at 4 °C for 12 h to complete the assembly of artificial cellulosomes. The extracellular surface displaying proteins were analyzed with immunological response by immunofluorescence microscopy and flow cytometry. We detected each protein element of minicellulosome by labeling special peptide tags with corresponding antigens. Incubated yeast cells were harvested by centrifugation at 3000 rpm for 5 min. The cells were resuspended with 250 μl rabbit anti-6 × His tag antibody or mouse anti-Xpress tag antibody (1:1000 dilution). Added Alexa Fluor^®^ 488-conjugated Goat anti-Mouse IgG (H + L) and Alexa Fluor^®^ 647-conjugated goat anti-Rabbit IgG (H + L) after twice washes. Whole cell fluorescence images of the surface-displayed components were detected with a fluorescence microscope (LSM 780, Germany). The percentage of labeled cells was estimated using CSampler TM Plus (BD) (Tsai et al. 2009; Wen et al. 2010).

### Ethanol batch fermentation

Cellulosic ethanol batch fermentation was conducted to test the ethanol production of the engineered CBP–hemicellulosome yeast consortium. 50 g/l steam-exploded P. purpureum or 10 g/l of birchwood xylan (β-D-xylopyranose) was used as the carbon source with 100 mL of sodium acetate buffer (pH 4.8, 50 mM) in 250 mL Erlenmeyer flasks. Novozymes Cellic CTec2 with an activity loading of 15 FPU/g cellulose was utilized. The prehydrolysis procedure was started up once enzyme addition under the condition of 50 °C, 200 rpm for 6 h. When the temperature dropped to 35 °C with agitation at 90 rpm under microaerophilic conditions, 1.0 g/l (wet weight) of the yeast cells harvested from logarithmic phase cultures were added into the reaction system. Different recombinant strains were fed in equal proportion. The inoculation was referred to as the beginning time of SSF. Total sugar and ethanol in the broths were obtained from triplicate experiments and measured according to our previous work (Wang et al. 2014). Ethanol yield was calculated on the basis of total sugar consumed. All the last reported data was the triplicate's average value, and the standard deviation was calculated as a measurement error. *T* test was used to analyze the significance of the results.

## Results and discussion

### Di-CRISPR design for XR-XDH pathway

We tested Di-CRISPR at certain delta-targeting guide RNA to achieve multicopy chromosomal integration with high efficiency of reductase–xylitol dehydrogenase pathway in *S. cerevisiae*. Two xylose metabolic genes, *XYL1* and *XYL2*, were introduced into δ-integration of yeast WA1 genomes simultaneously using CRISPR/Cas9. As expected, multicopy genome integration of heterologous xylose metabolic pathway was observed to a remarkable increase with elevated DNA levels.

**Fig. 1:**
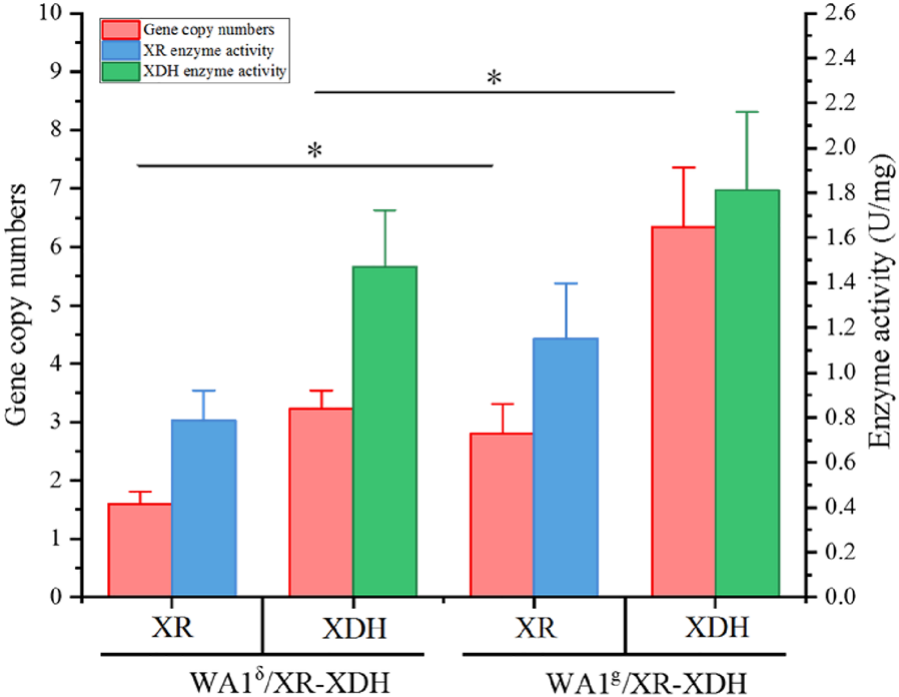
Gene copy number and enzyme activity of the recombinant yeast strains with δ integration and Di-CRISPR, respectively. The values and error bars represent average readings and standard deviations for 3 qPCR reactions. *Indicates statistically significant differences between Di-CRISPR and conventional delta integration populations (student's *t* test, *p* < 0.05)

The highest copy number of *XYL1* and *XYL2* for δ-integration through CRISPR/Cas9 mediation was 2.8 and 6.3, respectively, whereas it was 1.6 and 3.2 for conventional delta integration. It demonstrated that the new replicative CRISPR/Cas9-mediated targeted δ-integration was effective in genome integration. As a result, the transformants with optimized protein expression were subsequently selected by combining enzyme activity analysis. As shown in Fig. 1, the yeast strain WA1^g^/XR-XDH with higher copy-numbers than WA1^δ^/XR-XDH exhibited higher enzyme activities, thus suggesting the optimized regulation of xylose metabolic flux in *S. cerevisiae* is proportional to the efficiency enhancement of multi-copy chromosomal integration and multiplex genome engineering approach.

### Construction of cellulosome-producing CBP reaction system

**Fig. 2:**
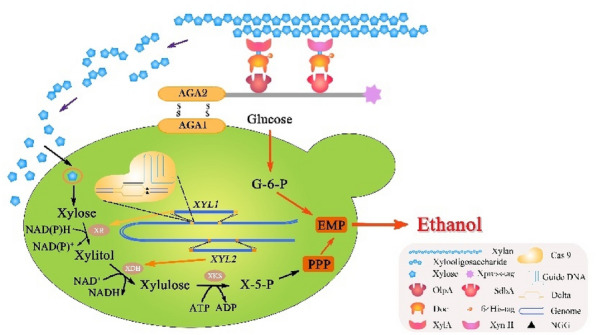
Schematic diagram of the designer cellulosome self-assembled on the cell surface of xylose-utilizing recombinant S. cerevisiae strain WA1^g^ under the optimization of δ-integration CRISPR Cas9

We designed an artificial hemicellulosome by mimicking the natural mechanism on the recombinant xylose-utilizing strain WA1^g^/XR-XDH. Although some functional cellulases are already genetically immobilized on *S. cerevisiae* for combinatorics and on-site enzyme loading, “chimeric hemicellulases and minihemicellulosome” have few been employed in “arming yeast” strategy for simultaneous hydrolysis and fermentation. As shown in Fig. 2, the displaying scaffoldin served as the integrating carrier for hemicellulase recruitment and was docked on WA1^g^/XR-XDH cell surface via a-agglutinin anchor system. The scaffoldin setup can enhance the quality of multi-enzymatic co-localization and provide flexibility for the adaptive assembly of chimeric components. Hence, the cellulosome-producing engineered microorganism enables us to achieve the synergistic reaction of CBP system on biomass accessibility by consolidating the simultaneous lignocellulose hydrolysis and available sugar co-fermentation. Moreover, considering the metabolic burden of host cell imparted by mini-cellulosome with the complex structure, we devise a CBP-enabling *S. cerevisiae* consortium, in which every engineered yeast strain could secrete or display different assembly component to be adaptively assembled on the surface of the scaffoldin-displaying yeast cell.

### Secreted expression and enzyme assay

**Fig. 3:**
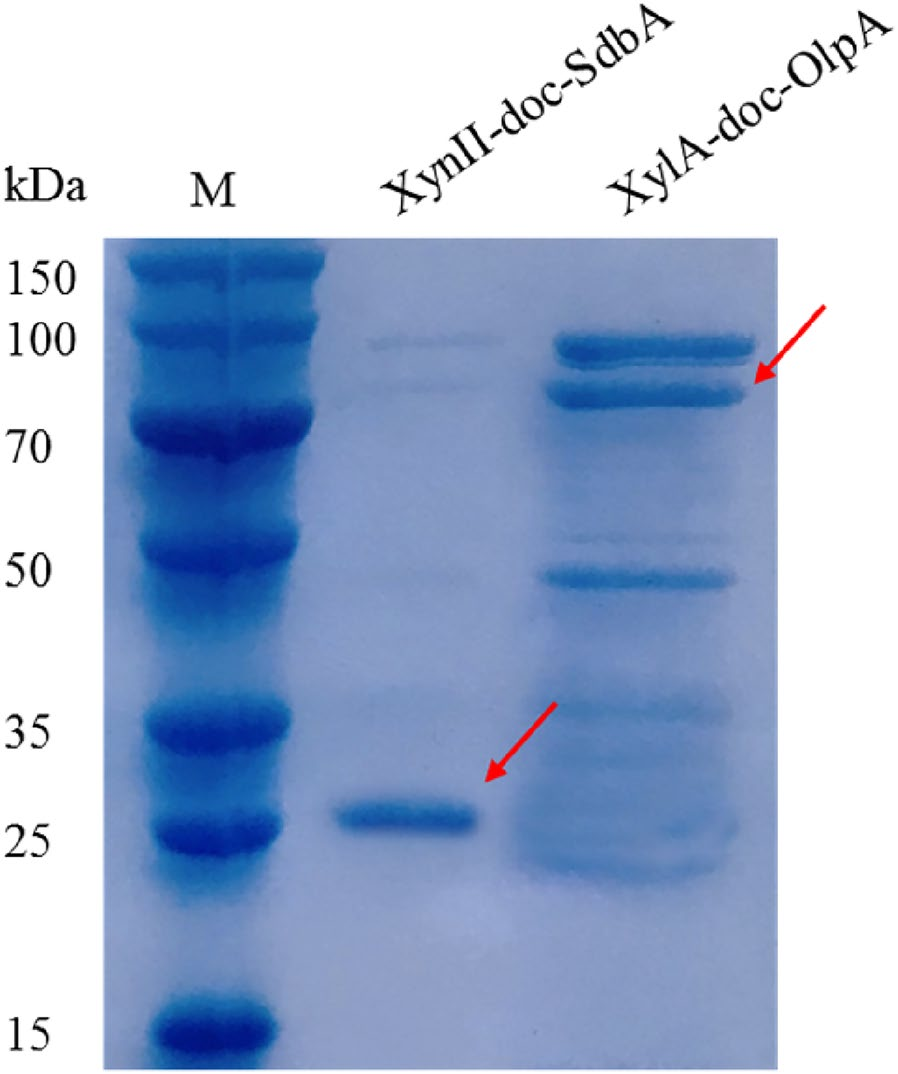
Analysis protein secreted expression by SDS–PAGE gel staining with Coomassie bright blue

We constructed recombinant yeasts for expressing dockerin-fused endoxylanase or xylosidase. The secreted expressions of full-length XynII-doc-SdbA (29 kDa) and XylA-doc-OlpA (92 kDa) were detected by SDS–PAGE (Fig. 3). On the basis of docking of catalytic modules into the displaying scaffoldin, the functional enzyme activities were also determined. As shown in Table 3, two types of xylanases were examined in the cell culture supernatants of WA1^g^/XA and WA1^g^/Xn, respectively. The WA1^g/^NC strain with empty PYD1 plasmid was used as a control. Each recombinant strain showed enzymatic activity of xylanase according to the xylooligosaccharides tested in the culture.

**Table 3:** Enzyme activities of xylanases

Strain	Substrate	Enzyme activity (U/ml)
WA1^g^/NC	1% Xylan	0.14 ± 0.04
	0.8% *p*NPX	0.09 ± 0.02
WA1^g^/XA	1% Xylan	3.13 ± 0.29
WA1^g^/Xn	0.8% *p*NPX	3.46 ± 0.34

### Functional localization and assembly of artificial cellulosome

**Fig. 4:**
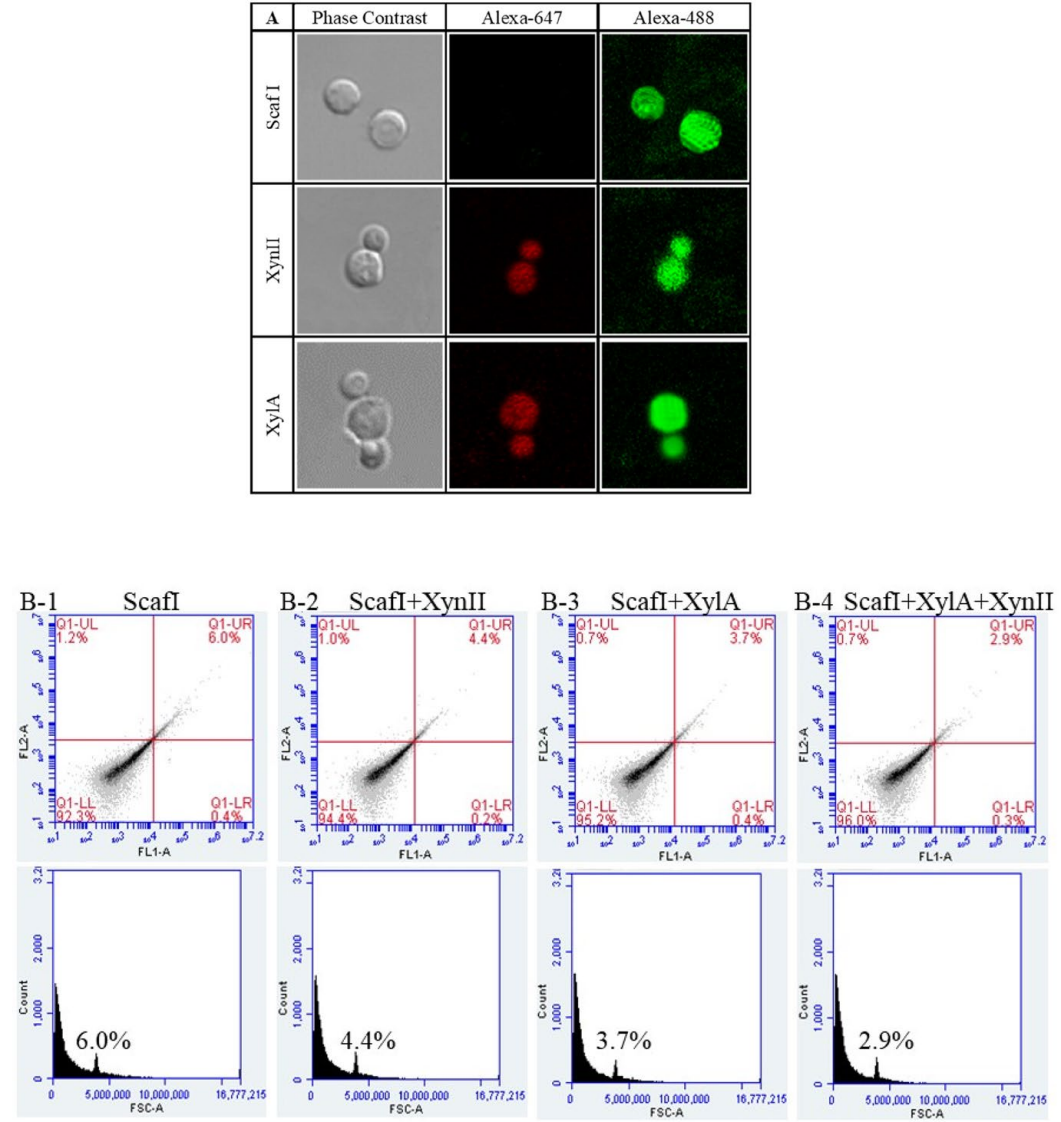
Functional assembly of yeast surface-displayed cellulosome. **A** Immunofluorescence micrographs of yeast whole cells displaying the anchoring scaffoldin and docked with enzymes. Cells were probed with anti-Xpress or anti-6 × His fluorescently stained with a Goat anti-Mouse IgG (H + L) conjugated with Alexa Fluor 488, Goat anti-Rabbit IgG (H + L) conjugated with Alexa Fluor 647, respectively. **B** Flow cytometric analysis of positive cells displaying different cellulosomal component

With the successful expression of catalytic units, the capability of the artificial cellulosome to recruit chimeric enzyme components onto the displaying-scaffoldin via the specific binding interaction between cohesin and dockerin was further illustrated by immunofluorescence examination. The accessibility test of scaffoldin and dockerin-containing enzyme protein targeting with different epitope tags was performed. Here, we detected the fluorescence of WA1^g^/ScafI and consortiums incubated with separate enzyme-secreting strains and WA1^g^/ScafI to verify the assembly. As shown in Fig. 4A line 1, the green immunofluorescent labeling of ScafI was detected using goat anti-mouse IgG (H + L) conjugated with Alexa Fluor 488. This result confirmed that the displaying scaffoldin has successfully been anchored on the host cell surface. In Fig. 4A line 2 and line 3, bright red fluorescence and green fluorescence at 647 nm and 488 nm were detected on the surface of cultured yeast cells, indicating that the two types of dockerin containing hemicellulases were already integrated into the scaffoldin. Finally, the relative assembling levels of these recruited enzymes were quantitatively assessed via testing the corresponding epitope by flow cytometry. We examined the effects of one enzyme assembly and two enzymes assembly simultaneously. As shown in Fig. 4B (upper right quadrant), positive populations were detected for all recombinant strains, which suggested that whole compositions of minicellulosomal architecture were successfully displayed on the WA1^g^ cell surface and achieved the desired results. However, compared to docking a single cellulosomal component, it should be noted that co-docking the scaffoldin and chimeric enzymes continuously on the cellulosome structure resulted in an obvious decrease in the positive populations (Fig. 4B), which reflected a reduced assembly efficiency caused by steric hindrance. Therefore, we might need to improve the minicellulosomal machinery and organization by designing scaffoldin linker between the protein modules and the construction of carbohydrate binding modules (CBMs) to target appended catalytic units to the substrate.

### Xylan hydrolysis and ethanol fermentation

**Fig. 5:**
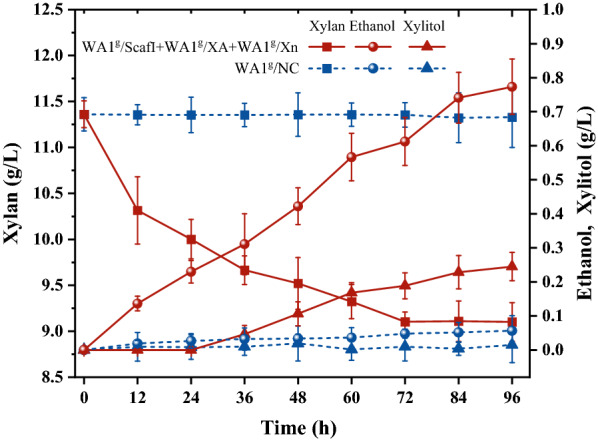
Time profiles of hydrolysis and ethanol production from birchwood xylan using two chimeric enzymes docking on the designer cellulosome

To test the fermentability of yeast consortium, the recombinant yeast strains WA1g/ScafI, WA1^g^/XA, and WA1^g^/Xn were added in equal proportion to simultaneous hydrolysis and fermentation from birchwood xylan, which contains > 90% xylose residues. Figure 5 shows the time course of xylan utilization and ethanol production during the consolidated bio-processing process. An amount of hemicellulosic substrate was ceaselessly consumed by the yeast consortium within 96 h with a slow but steady consumption rate of 6 mg/l/h. Meanwhile, the decrease of the xylan concentration was consequently accompanied by the increase of ethanol titer. The highest ethanol concentration reached 0.61 g/l with 67.01% theoretical yield based on the consumed xylan. The results demonstrated the functional activity of chimeric xylanases and the applicability of CBP-enabling yeast consortium in direct hemicellulose-to-ethanol conversion.

### SSCF performance from steam-exploded P. purpureum

**Fig. 6:**
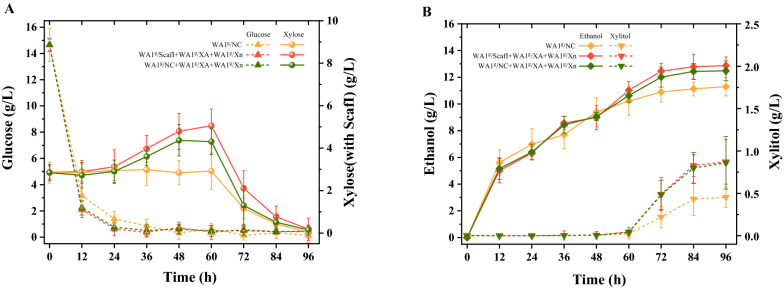
Time profiles of lignocellulose hydrolysis (**A**) and ethanol production (**B**) from steam-exploded *P. purpureum* after 6 h pre-hydrolysis using recombinant yeast consortium

We previously conducted a fed-batch operation mode for cellulosic ethanol production (Wang et al. 2014). We followed the process to SSCF from steam-exploded *P. purpureum* in this work, involving the adaption of yeast culture and the pre-hydrolysis prior to inoculation. The functionality of yeast consortium with a synergistic combination of endoxylanase and xylosidase in simultaneous saccharification holocellulose and co-fermentation ethanol were further studied. As presented in Fig. 6 and Table 4, as the initial carbon source for the growth and metabolism of recombinant yeast strains, an amount of glucose was released after 6 h-prehydrolysis of solid liquefactions. However, the subsequent glucose concentration during the fermentation process maintained a considerably low level, suggesting the robust metabolic capacity and fermentation property of yeast consortium. While produced xylose derived from hemicellulose hydrolysis was accumulated before 60 h due to catabolite repression, it was not used until glucose was depleted. It is worth noting that the amount of released xylose additionally affording by the CBP yeast consortium led to significant differences in substrate utilization and maximum ethanol production between the experimental group and control group (*p* < 0.01). A total of 4.87 g/l xylose was consumed with the accumulated xylitol at a yield of 0.18 (g/g xylose) during 96 h of fermentation. Although xylitol was produced unavoidably from xylose under the influence of redox imbalance in xylose reductase–xylitol dehydrogenase pathway, which was directly caused by different cofactor specificities of NAD^+^-dependent XDH and NADPH-preferring XR in recombinant *S. cerevisiae*, the maximum ethanol concentration achieved 12.88 g/l with the maximal cellulose conversion rate of 91.21% and hemicellulose conversion rate of 86.41% (Table 4). Implementing high-solids fermentation for high-titer ethanol production to facilitate downstream separation would be necessary for further work.

**Table 4:** Summaries of SSCF performance of engineered yeast consortium

Engineered yeast consortium	Maximum ethanol concentration** (g/l)	Theoretical ethanol yield^a^ (%)	Substrate consumption rate (g/l/h)^d^	Cellulose conversion rate^b^ (%)	Hemicellulose conversion rate^c^ (%)
WA1^g^/ScafI + WA1^g^/XA + WA1^g^/Xn	12.88 ± 0.64	84.87 ± 0.26	0.31 ± 0.01	91.21 ± 0.89	55.25 ± 0.37
WA1^g^/NC + WA1^g^/XA + WA1^g^/Xn	12.48 ± 0.74	86.04 ± 0.34	0.30 ± 0.02	90.14 ± 0.71	49.37 ± 0.44
WA1^g^/NC	11.47 ± 0.70	86.92 ± 0.32	0.27 ± 0.01	86.41 ± 0.59	-

^a^$${\text{Ethanol}}\,{\text{yield}}\,\left( \% \right) = \frac{{{\text{produced}}\,{\text{ethanol}}\,\left( {\text{g}} \right)}}{{{\text{consumed}}\,{\text{glucose}}\,\left( {\text{g}} \right) \times 0.51 + {\text{consumed}}\,{\text{xylose}}\left( {\text{g}} \right) \times 0.46}} \times 100\%$$

^b^$${\text{Cellulose}}\,{\text{conversation}}\,{\text{yield}}\left( \% \right) = \frac{{{\text{Cellulose}}_{{\text{s}}} \left( {\text{g}} \right) - {\text{Cellulose}}_{{\text{R}}} \left( {\text{g}} \right)}}{{{\text{Cellulose}}_{{\text{s}}} \left( {\text{g}} \right)}} \times 100\% \,\left( {{\text{Cellulose}}_{{\text{s}}} {\text{:cellulose}}\,{\text{from}}\,{\text{total}}\,{\text{substrate;}}\,{\text{Cellulose}}_{{\text{R}}} {:}\,{\text{cellulose}}\,{\text{from}}\,{\text{fermentation}}\,{\text{residues}}} \right)$$

^c^$${\text{Hemicellulose}}\,{\text{conversation}}\,{\text{yield}}\left( \% \right) = \frac{{{\text{Hemicellulose}}_{{\text{s}}} \left( {\text{g}} \right) - {\text{Hemicellulose}}_{{\text{R}}} \left( {\text{g}} \right)}}{{{\text{Hemicellulose}}_{{\text{s}}} \left( {\text{g}} \right)}} \times 100\% \,\left( {{\text{Hemicellulose}}_{{\text{s}}} :{\text{hemicellulose}}\,{\text{from}}\,{\text{total}}\,{\text{substrate}};\,{\text{Hemicellulose}}_{{\text{R}}} :{\text{hemicellulose}}\,{\text{from}}\,{\text{fermentation}}\,{\text{residues}}} \right)$$

^d^$${\text{Substrate}}\,{\text{consumption}}\,{\text{rate}}\,\left( {\text{g/l/h}} \right) = \frac{{{\text{Consumed}}\,{\text{cellulose}} \times 1.1\,\left( {\text{g/l}} \right) - {\text{Consumed}}\,{\text{hemicellulose}} \times 1.136\left( {\text{g/l}} \right)}}{{96\left( {\text{h}} \right)}}$$

^**^Indicates statistically significant differences between experimental group and control group (*T* test, *p* < 0.01)

As the accessory enzyme for hydrolysis and the supplement of commercial cellulases, xylanase could alter the macromolecular structure of lignocellulosic material and thus improve cellulose enzymatic hydrolysis by increasing access of cellulases to the substrate. Some xylan-degrading yeast strains by displaying or expressing hemicellulases in xylose-utilizing host cells have been observed previously, while almost all of them did not involve the engineering xylanolytic minicellulosome for CBP whole-cell biocatalyst (Katahira et al. 2004; Sakamoto et al. 2012). Sun et al. successfully designed the cellulosomal module consisting of three xylanases, but it appeared to show a relatively low resulting co-fermentation performance (Sun et al. 2012). In addition, a xylose/glucose co-fermentation process by co-culture of cellulose-utilizing recombinant *Saccharomyces cerevisiae* and xylan-utilizing recombinant *Pichia pastoris* was developed (Zhang et al. 2017). However, the co-culture of different engineering microbes simulating the unnatural state of microbial community may cause potential metabolic competition. Therefore, further evaluation about the feasibility of a co-culture reaction system for direct simultaneous saccharification and co-fermentation would be necessary. In this work, the holocellulose-to-ethanol conversion from pretreated perennial C4 grass was achieved on the basis of incorporating the functional mini-hemicellulosome into the xylose-utilizing yeast strain under the optimization of δ-integration CRISPR Cas9. The structure of the hemicellulosome and its responsible function in SSCF bioreaction system of pretreated lignocellulose were further evaluated with the addition of commercial cellulase.

In general, it is a practical benefit in integrating catalytic units of hemicellulase into the multi-enzymatic formation of the CBP system for enhancing biomass accessibility and cellulosic ethanol co-fermentation of pentose and hexose. On the other hand, besides the potential enzyme proximity synergy, the enhanced hydrolysis rate of holocellulose observed for the functional minicellulosome might be due to an overall effect caused by the yeast cell surface-display system, secretion efficiency of exogenous enzyme, minicellulosomal machinery and the like. Further studies are, therefore, required to improve the elaborated structural organization of designer hemicellulosome for the ideal consolidated bioprocessing based on simultaneous holo-cellulose saccharification and fermentation.

## Conclusions

A designer hemicellulosome was successfully adaptive-assembled on the cell surface of xylose-utilizing *S. cerevisiae* under the optimization of δ-integration CRISPR Cas9. The surface-displaying minicellulosome consisted of the dockerin-containing xylosidase XynII and endoxylanase XylA without carbohydrate binding modules. The amount of released xylose additionally affording by CBP yeast finally led to significant differences in substrate utilization and maximum ethanol production between experimental and control groups. Despite the accumulation of xylitol, the maximum ethanol concentration achieved 12.88 g/l during 96 h with the maximal cellulose conversion of 91.21% and hemicellulose conversion of 55.25%. These results demonstrate the applicability of designer hemicellulosome toward lignocellulose saccharification and co-fermentation in *S. cerevisiae* and the importance of the minicellulosome-producing CBP whole-cell biocatalyst for cost-effective biofuel production.

### Supplementary Information

**Additional file 1: Table S1. **Primers used for cloning. **Table S2. **Primers used for qPCR. **Figure S1.** A schematic representation of plasmid construction. **Figure. S1A.** pDi-g1 plasmid for δ-integration CRISPR–Cas9. **Figure. S1B. **Surface displaying plasmid for anchoring scaffoldin. **Figure. S1C. **Secretion expression plasmids for two types of xylanases.

## Data Availability

All data sets used and analyzed are available on reasonable request.

## Abbreviations

SSCF: Simultaneous saccharification and co-fermentation
CBP: Consolidated bioprocessing
XR: Xylose reductase
XDH: Xylitol dehydrogenase
Di-CRISPR: CRISPR–Cas9-mediated δ-integration
DSBs: Double stranded breaks
PGK: Phosphoglycerate kinase Promoter
XylA: Xylanase
XynII: Xylosidase
αMF: α-Mature factor signal peptide
PBS: Sodium phosphate buffer
DNS: 3,5-Dinitrosalicylic acid
PASC: Phosphoric acid swollen cellulose
*p*NPX: 4-Nitrophenyl β-D-xylopyranoside
