# Supplementary material for: An accessory enzymatic system of cellulase for simultaneous saccharification and co-fermentation
Source: Bioresour Bioprocess. 2022 Sep 19;9(1):101. doi: 10.1186/s40643-022-00585-5 (PMC10991206; doi:10.1186/s40643-022-00585-5)

**Additional file 1**

**Table S1** Primers used for cloning.

| Plasmid and DNA | Sequence (5’→3’) |
| --- | --- |
| pYD1-ScafI | *olpa* F: GAGGATCCACAAACACCATTGAAATAA  *olpa* R: GCTCGAGGCTTTATCTGCCTCCGGAGCGGATG |
|  | *sdba* F: CATCCGCTCCGGAGGCAGATAAAGCCTCGAGC  *sdba* R: CTGCGGCCGCTTATGCAGGCGAAGGCGTC |
| pYD1-XynII | *xynii* F: GAGGATCCAAAAGACGCCAGACGATTCAGC  *xynii* R: TGGAGCCTGTGAAGTGCTGACGGTGATGGA |
|  | *doc-sdba* F: TCCATCACCGTCAGCACTTCACAGGCTCCA  *doc-sdba* R: AGCTTTGTTTAAACTTACTGTGCGTCGTAA |
| pYD1-XylA | *xyla* F: GAGGATCCAAAAGACAAGCAAACCAAAGC  *xyla* R: CATTAAGCACAACAGGTTGCGGCGCAATCAAC |
|  | *doc-olpa* F: GTTGATTGCGCCGCAACCTGTTGTGCTTAATG  *doc-olpa* R: AGCTTTGTTTAAACTTATATAGTTATAAGTCC |
| 5’ Delta homologous arms | 5’-*δ* F: GCCGCGTGTTGGAATAGAAATCAACTATC |
|  | 5’-*δ* R: GAGGAAGCTGAAACGCAATATTTTAGATTCCTGACTTC |
| 3’ Delta homologous arms | 3’-*δ* F: AATACTACTCAGTAATAAAAAATGATGATAATAATAT |
|  | 3’-*δ* R: CGGCCATGAGAAATGGGTGAATG |
| PGK | *pgk* F: GAGGAAGCTGAAACGCAATATTTTAGATTCCTGACTTC |
|  | *pgk* R1: CAACTTAATAGAAGGCATCGTTTTGTTTTATATTTGTTGTA  *pgk* R2: GGAAGGGTTAGCAGTCATCGTTTTGTTTTATATTTGTTGTA |
| XR | *xr* F: ACAACAAATATAAAACAAAACGATGCCTTCTATTAAG |
|  | *xr* R: GTTATCAGATCAGCGGGTTTAAACTTAGACGAAGATAGGAATC |
| XDH | *xdh* F: TACAACAAATATAAAACAAAACGATGACTGCTAACCCTTCC |
|  | *xdh* R: GTTATCAGATCAGCGGGTTTAAACTTACTCAGGGCCGTCAATG |
| MATT | *MATT* F1: GATTCCTATCTTCGTCTAAGTTTAAACCCGCTGATCTGATAA  *MATT* F2: CATTGACGGCCCTGAGTAAGTTTAAACCCGCTGATCTGATA |
|  | *MATT* R: TATTATTATCATCATTTTTTATTACTGAGTAGTAT |
| gRNA1 | gRNA1-F: CCAAAACGCAAGGATTGATAATGTAATGTTTT |
|  | gRNA1*-*R: CTCTAAAACATTACATTATCAATCCTTGCGTT |

**Table S2** Primers used for qPCR.

| Target Gene | Sequence (5’→3’) |
| --- | --- |
| XYL1 | qX1-F: GAGGTGCTACCATCAAGCCATCTG |
|  | qX1-R: CGAACGAAGAGTAAGCGGTGACAG |
| XYL2 | qX2-F: CTTTGGTGCTGGTCCTGTTGGTC |
|  | qX2-R: CGGTCTTGGAGTTGAAGGTGTGAG |
| ALG9 | qALG9-F: GCCGTCTACGAGCAATTTTC |
|  | qALG9-R: ATCTGGCAGCAGGAAAGAAC |

**Figure S1.** A schematic representation of plasmid construction.

**Fig. S1A.** pDi-g1 plasmid for δ-integration CRISPR-Cas9.


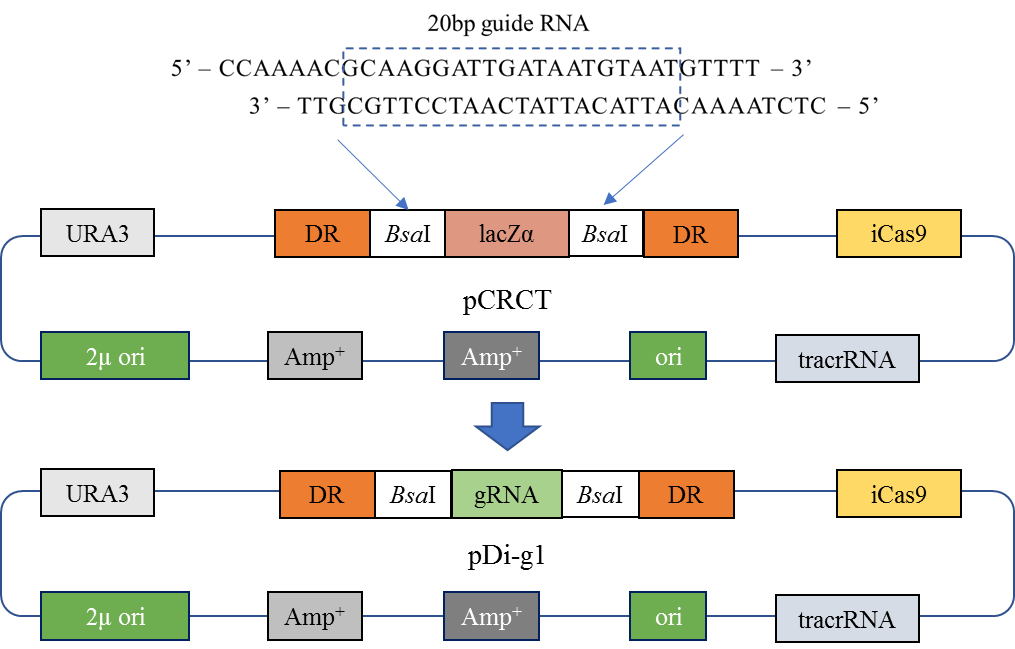


**Fig. S1B.** Surface displaying plasmid for anchoring scaffoldin.


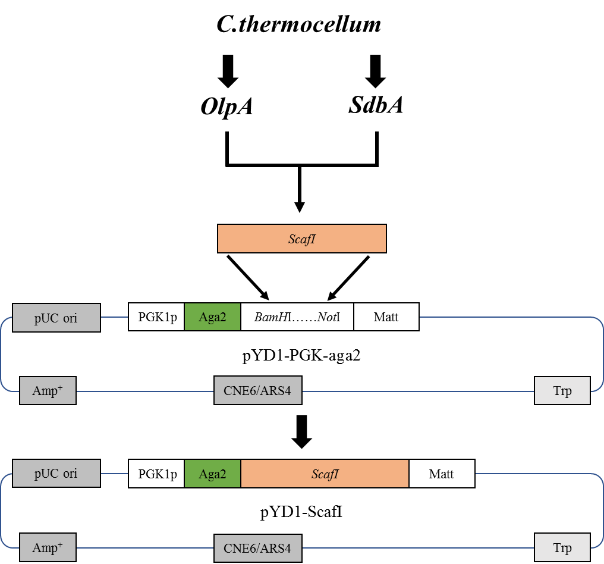


**Fig. S1C.** Secretion expression plasmids for two types of xylanases.


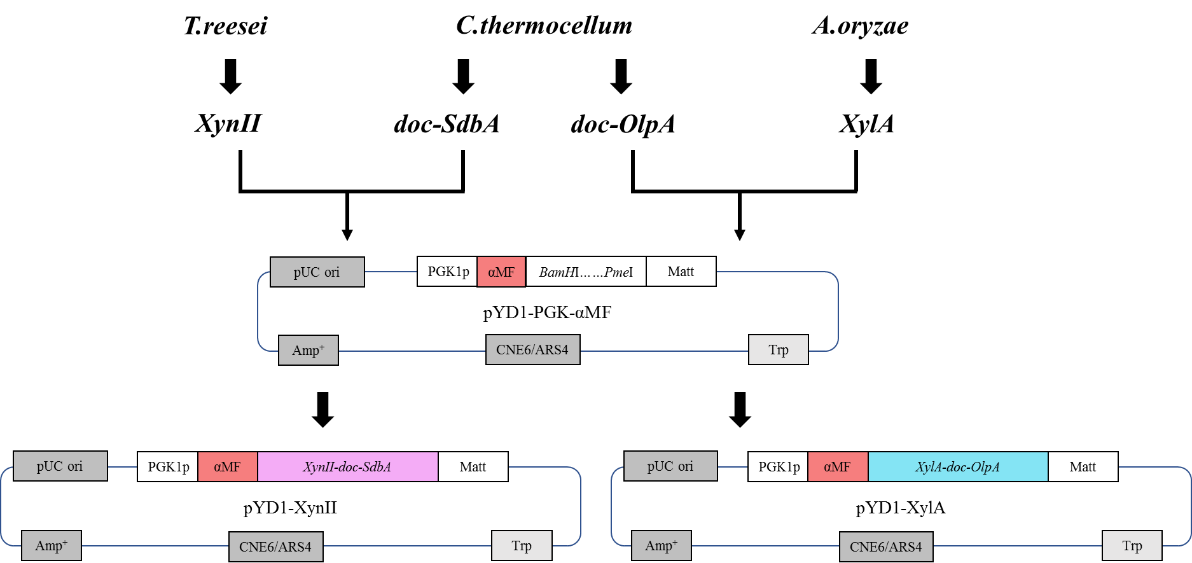

Supplement: Supplementary file 1 — Additional file 1: Table S1. Primers used for cloning. Table S2. Primers used for qPCR. Figure S1. A schematic representation of plasmid construction. Figure. S1A. pDi-g1 plasmid for δ-integration CRISPR–Cas9. Figure. S1B. Surface displaying plasmid for anchoring scaffoldin. Figure. S1C. Secretion expression plasmids for two types of xylanases. [file 40643_2022_585_MOESM1_ESM.docx]
